# Case report: A novel mutation in *TRPS*1 identified in a Chinese family with tricho-rhino-phalangeal syndrome I: A therapeutic challenge

**DOI:** 10.3389/fped.2022.990230

**Published:** 2022-11-18

**Authors:** Qi Huang, Cheng Jiang, Jiazhong Sun, Junli Xue, Victor Wei Zhang

**Affiliations:** ^1^ Department of Endocrinology, Zhongnan Hospital of Wuhan University, Wuhan, China; ^2^ Emergency Center, Zhongnan Hospital of Wuhan University, Wuhan, China; ^3^ Department of Endocrinology, The Second Clinical Medical College, Yangtze University, Jingzhou, China; ^4^ Department of Clinical Genetics, AmCare Genomics Lab, Guangzhou, China

**Keywords:** tricho-Rhino-Phalangeal syndrome, *TRPS*1 gene, deletion mutation, short stature, sparse hair

## Abstract

Tricho-rhino-phalangeal syndrome (TRPS) is a rare autosomal dominant malformation caused by mutations involving the *TRPS*1 gene. Patients with TRPS exhibit distinctive craniofacial and skeletal abnormalities. This report presents three intra-familial cases with *TRPS*1 gene mutations that showed the characteristic features of TRPS. A 13-year-old boy was admitted to Department of Endocrinology for the evaluation of short stature. Physical examination revealed that the boy had thin sparse hair, pear-shaped nose, protruding ears, small jaw and brachydactyly. A survey of his family history indicated that the boy's sister and mother shared the same clinical features. Radiological techniques demonstrated a different degree of skeletal abnormalities in these siblings. Next-generation sequencing and quantitative PCR were performed and showed a novel deletion mutation in exons 3–5 in the three familial cases, confirming the diagnosis of TRPS I. The healthy father did not carry the deletion mutation. Currently, there was no specific therapy for TRPS I; however, genetic consultation may be useful for family planning

## Introduction

First reported by Giedion in 1966 (1), Tricho-rhino-phalangeal syndrome (TRPS) is a rare heritable congenital or sporadic disorder characterized by typical craniofacial features and noticeable skeletal abnormalities, especially of phalanges, metacarpals and metatarsal bones (2–4).

Based on clinical characteristics and genetic analysis, TRPS is distinguished into three subtypes: TRPS I (OMIM 190350), known as Giedion syndrome, have distinct clinical manifestations that often correspond to distinct mutations or haploinsufficiency in the *TRPS*1 gene (5). Moreover, TRPS II (OMIM 150230), also named Langer-Giedion syndrome (LGS), is caused by a contiguous gene deletions involving both *TRPS*1 and *EXT*1 (6, 7). TRPS II differs from TRPS I by the presence of multiple exostoses and intellectual disability (6). TRPS III (OMIM 190351) is also associated with *TRPS*1 mutations. Besides typical TRPS feature, TRPS III cases have more severe skeletal malformations (7).

**Figure 1:**
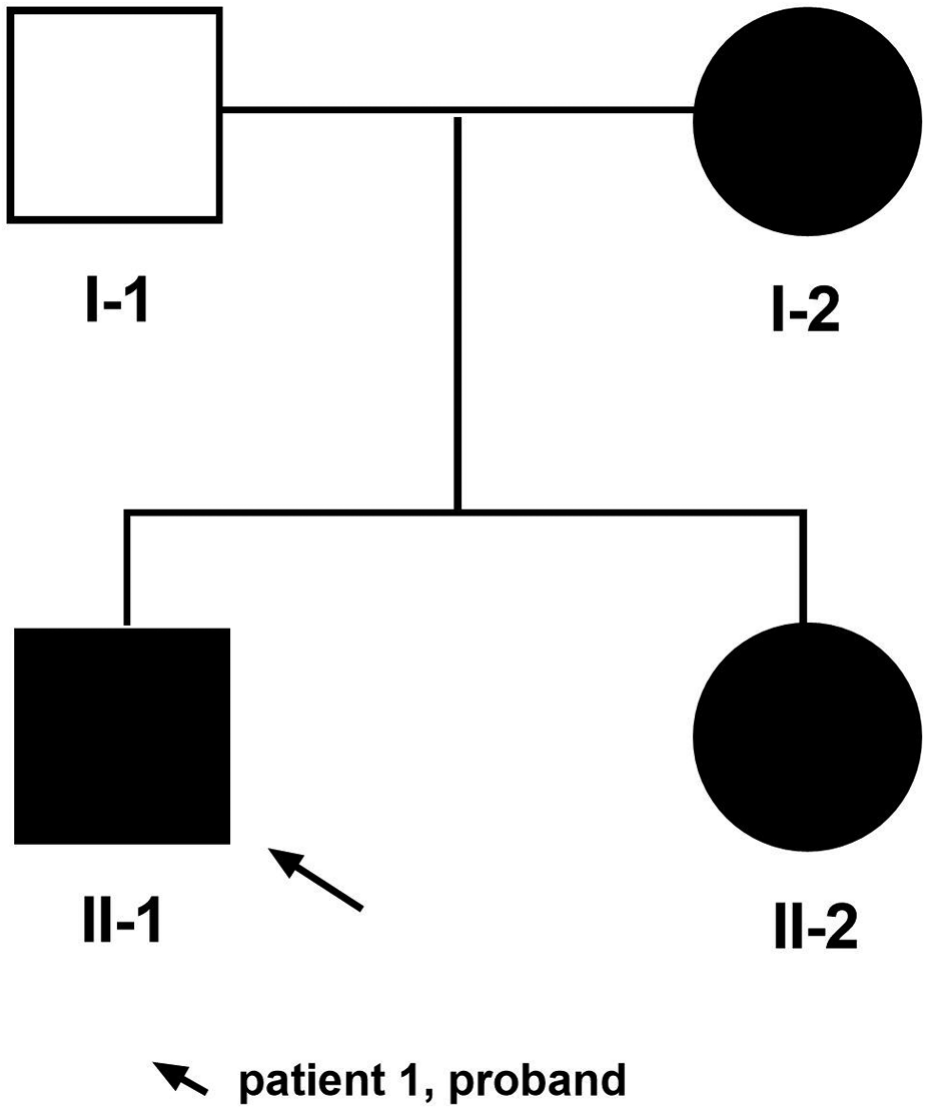
Pedigree of family. The arrow indicates patient 1 as the proband.

Herein, we describe a Chinese Han family with three TRPS I cases caused by a novel deletion mutation in the *TRPS*1 gene involving exons 3–5 (Figure 1).

## Patients and clinical evaluation

### Case 1

A 13-year-old boy, proband, was first admitted to our Endocrinology Department for evaluation of his short stature. He was born after a full term pregnancy and normal delivery, as the second child in non-consanguineous family. His parents reported that his birth weight and length was normal but gradually developed short stature upon birth. In addition, he often suffered from respiratory infections and his tonsils were removed. However, no intellectual impairment was observed.

**Figure 2:**
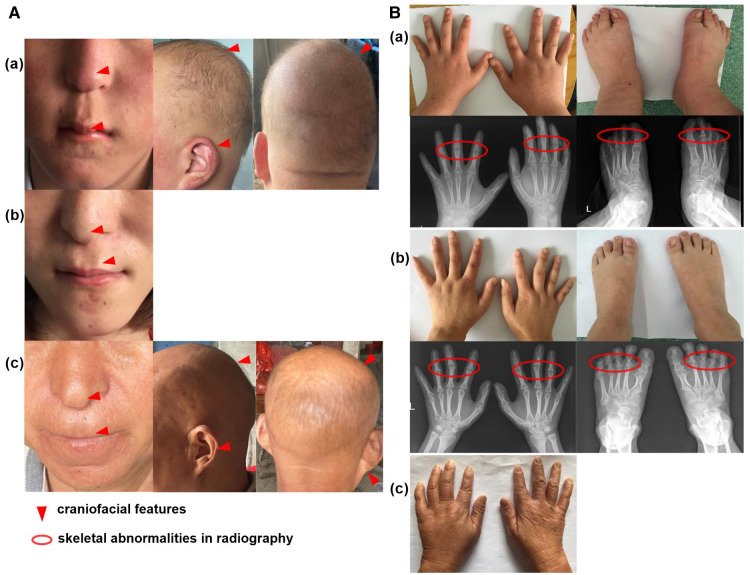
Clinical feature of the family cases. (**A**) The craniofacial feature of proband (a), his sister with wig (b) and mother (c); (**B**) The skeletal abnormalities of proband (a), his sister (b) and mother (c).

Upon admission, a routine examination revealed that the boy's weight was 81 kg (>97th percentile) and his standing height was 152.2 cm (3rd percentile). Pubertal development was normal. Another prominent dysmorphic feature included markedly thin and sparse scalp hair, protruding ears, a bulbous pear-shaped nose and a long philtrum with a thin upper lip [Figure 2A(a)].

Laboratory tests showed that serum levels of calcium, inorganic phosphate, alkaline phosphatase, free T4, TSH, PTH, corticosteroid and insulin-like growth factor 1 (IGF-1) were normal. The karyotype was 46, XY. Further extremity and radiological examinations showed brachydactyly of fingers and toes [Figure 2B(a)].

### Case 2

The patient was a 23-year-old girl, the elder sister of patient 1. She showed similar features to her brother, with a height of 146 cm (<3rd percentile). She was almost bald and declined to take off her wig [Figure 2A(b)]. She showed a brachydactyly with obvious clinodactyly, a deviation of the forefinger, middle fingers and ring fingers bilaterally. Radiography revealed distortion of the proximal middle phalanges on the second, third and fourth fingers bilaterally. She also showed a skeletal malformation on second through fifth proximal phalanx on both feet [Figure 2B(b)].

### Case 3

The mother of the siblings, with a height of 140 cm (<3rd percentile), presented with sparse scalp hair and a nose with a bulbous tip [Figure 2A(c)]. She also showed bone abnormalities on her hands [Figure 2B(c)], but she declined further laboratory tests and radiological examinations.

## Molecular analysis

Genomic DNA from the proband and his family members was extracted from peripheral blood samples. A custom-designed Medical Exome Sequencing (MES, AmCare Genomic Lab), including target region capture of more than 5,000 phenotype-related genes contained in the Online Mendelian Inheritance in Man (OMIM), was applied and was followed by next-generation sequencing (NGS, PE 150) on the Illumina platform (Illumina, Inc.). Alignment of the sequence to the reference human genome (hg19) was performed by NextGen (Softgenetics, LLC). Trio analysis including both SNV annotation and exome-based CNV identification was done by an in-house pipeline. Synonymous as well as common SNPs (MAF > 0.1% in gnomAD) were filtered out subsequently. All the candidate variants were further evaluated based on the ACMG guideline for SNV interpretation (8). A detailed protocol was described in a previous study (9).

The Candidate variant was validated by quantitative PCR (qPCR). Three pair primers of *TRPS*1 exon 3–5 were designed to amplify all exons of the deletion fragment as follows: TRPS1-EX3, 5′-TGAAACTGGGCTCAAACCTT-3′ (forward) and 5′-GGGG ACTCACTGGAGACAAA-3′ (reverse); TRPS1-EX4, 5′-CTGGTGGCCTCTGTACC ATT-3′ (forward) and 5′-ACAAAATA AAAGCTTCTCTCCCC-3′ (reverse); TRPS1-EX5, 5′- AGGAATCCCTTGGTTTCCAC -3′ (forward) and 5′-AGTCCGTCATACAC CCAAGC-3′ (reverse).

## Molecular Findings

**Figure 3:**
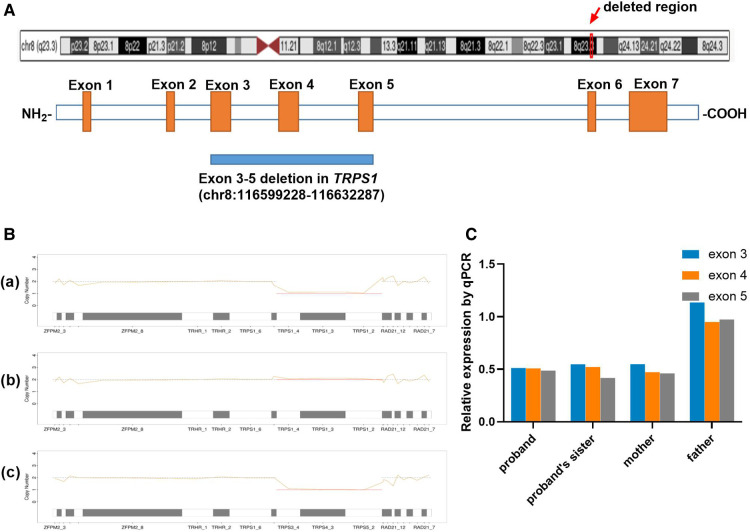
Genetic analysis. (**A**) Distribution of the muti-exon deletion in *TRPS*1 gene; (**B**) Copy number variation result of proband (a), his father (b) and mother (c) using NGS coverage depth data. The line charts present the copy number of 8q23 region for each family member, including *TRPS*1 and the gene's upstream and downstream locations. The proband and his mother were heterozygous for the deletion (×1) for exons 3–5 (CDS2-4), and his father was normal at this region. (**C**) qPCR results of all the family members. The proband, his sister and mother were carrying heterozygous deletion of exons 3–5 (×0.5), and his father was normal (×1).

Based on the MES trio analysis and qPCR validation, a small heterozygous deletion c.38−? _2700+? del within the *TRPS*1 gene (NM_014112.5) was identified (Figure 3A). It is segregated in all the patients of this family (proband, his elder sister and mother), and the healthy father did not carry the deletion (Figures 3B,C). This novel small deletion includes exons 3 to 5, and is not present in gnomAD database, HGMD or any peer-reviewed publication. It is predicted to disrupt the reading frame and undergo nonsense-mediated decay (NMD) resulting in an amino acid change (p.Asn13Lysfs*3) because of the multiple exons deletion. According to the ACMG guideline, this variant is classified as likely pathogenic.

## Discussion

*TRPS*1 was reported as the causal gene of TRPS I by Momeni et al. in 2000 (3). Haploinsufficiency is the known pathogenicity mechanism for the *TRPS*1 gene (7, 10). In a previous comprehensive study, deletion variants of *TRPS*1 have been reported in multiple cases, most of them are whole gene deletions that include exons 1 to 7 or large fragments deletions. Only one patient carrying a smaller (exon 2–6) deletion within the gene has been reported (2). The recurrence of variable sizes of fragment deletion suggests the structure complexity in this region.

We are reporting the second family carrying a small 3-exon deletion within the *TRPS*1 gene, which is predicted to disrupt the functional GATA motif of TRPS1. A mouse model study has revealed that a heterozygous knockdown of the GATA motif leads to hair and facial anomalies that overlap with findings of TRPS (11).

Our study also provides further evidence that structure variation is a common cause of TRPS. In this study, we used an optimized pipeline that combined both the SNV identification and NGS coverage depth data for CNV (even the small deletion/duplications) calls within one dataset, which proved to be a sensitive and cost-effective genetic analysis for the suspected TRPS patients, as well as for the better understanding of the genetic etiology of TRPS.

Definitive diagnosis of the disease is essential to perform timely therapeutic procedures. Nevertheless some alternative approaches have been tried for therapy of a few TRPS cases with mixed results. Short stature is a frequent clinical finding in affected individuals. How to improve their short stature is what these patients and their parents are most interested in. K Stagi (12) and Sarafoglou (13) described their TRPS I cases with or without growth hormone (GH) deficiency, and a remarkable increase in growth was observed through GH therapy in four cases. However, Naselli (14) reported another two TRPS I cases with poor growth, and showed no improvement in linear growth after a 1-year GH replacement therapy. In our study, the evaluation of GH-IGF-1 axis revealed that the boy did not have GH deficiency. His bone age was 15-year assessed through RUS-CHN radiographic atlas method, therefore he had no indications for GH treatment.

Sparse scalp hair is another major feature of TRPS patients. Their diffuse alopecia varies from normal hair to complete baldness (15), and the treatment option for alopecia remains unclear. Mi Soo Choi reported their experience in the medical treatment of a TRPS boy (15). In this case, neither topical minoxidil nor oral finasteride was effective in preventing the progression of alopecia or inducing hair growth. Finally, the patient's hairs started to re-grow at 4 months after hair transplantation operation. All of our three patients complained of hair loss and slow hair growth rate since their childhood. Compared to Mi Soo Choi's patient (15), whose occipital scalp hair had normal hair density and diameter, our patients' hair on the entire scalp was affected and tended to be thinner.

At present there is no special therapy for TRPS, even though alternative approaches were employed as GH replacement therapy for short stature and hair transplantation for baldness, the therapeutic results mixed. Therefore, genetic counseling may be useful for family planning.

## Data Availability

The datasets for this article are not publicly available due to concerns regarding participant/patient anonymity. Requests to access the datasets should be directed to the corresponding author.
